# Establishment of an out-of-hospital cardiac arrest registry in Penang, Malaysia

**DOI:** 10.1016/j.resplu.2026.101435

**Published:** 2026-08-06

**Authors:** K.E. Emily Leong, K.H. Benjamin Leung, Tomas Barry, Yuen Chin Leong

**Affiliations:** a School of Medicine, University College Dublin, Dublin, Ireland; b RCSI & UCD Malaysia Campus, Penang, Malaysia; c Department of Emergency Medicine, St. Michael’s Hospital, Unity Health Toronto, Toronto, Ontario, Canada; d Scottish Ambulance Service, Edinburgh, Scotland, UK; e School of Population Health, Royal College of Surgeons in Ireland, Dublin, Ireland; f Hospital Sungai Buloh, Ministry of Health Malaysia, Sungai Buloh, Selangor, Malaysia

## Abstract

**Introduction:**

In Penang, Malaysia, an out-of-hospital cardiac arrest (OHCA) registry was established in 2023 to facilitate systematic, sustainable collection and analysis of OHCA cases across the state. This paper describes the development of key data collection processes and presents a descriptive analysis of data captured during the first year of establishment.

**Methods:**

An online platform (myOHCA2.0) was developed to abstract data from existing pre-hospital and in-hospital electronic systems, capturing patient, event, response, and outcome characteristics in accordance with the Utstein template. OHCAs occurring between 1st April 2023 and 31st March 2024 were analysed.

**Results:**

A total of 5087 OHCA cases were included in the descriptive analysis. Patients were predominantly male (54.8%) with a median age of 73 years [IQR 61–83]. In total, 16.3% had a resuscitation attempt by EMS. A majority of patients were of either Chinese (52.2%) or Malay (33.0%) ethnicity, and most arrests occurred at home (92.8%). The median ambulance response time was 21 min [IQR 16–29]. Among EMS-attempted resuscitation cases, 47.2% were witnessed by laypersons, bystander CPR was provided in 43.5%, and an AED was applied in only 7.1%. Return of spontaneous circulation (ROSC) was achieved in 10.3% of this cohort, while 30-day survival was 1.3%.

**Conclusion:**

OHCA survival rates in Penang remain low despite bystander CPR being performed in nearly half of EMS-attempted resuscitation cases. Prolonged EMS response times and low rates of AED deployment represent targets for intervention.

## Introduction

Although data concerning out-of-hospital cardiac arrest (OHCA) had previously been collected in Penang, Malaysia, manual data abstraction without standardisation yielded limited information and was an unsustainable process.^1^ In response, the Penang State Health Department, in collaboration with the Malaysian National Institutes of Health, established the myOHCA2.0 platform to systematically collect OHCA data in the state of Penang from April 2023.

This paper describes the design of the Penang OHCA registry and provides a descriptive analysis of OHCAs recorded in the registry’s first year of operation as a means of establishing a foundation for quality improvement initiatives.

## Methods

### Study design

This study was a registry-based retrospective cohort study using data collected using myOHCA2.0 from 1st April 2023 to 31st March 2024 in Penang, Malaysia. The study was conducted in compliance with the Declaration of Helsinki, and ethics approval was obtained from the Medical Research Ethics Committee of the Ministry of Health, Malaysia (protocol #: KKM/NIHSEC/22-02543).

### Study setting

**Fig. 1:**
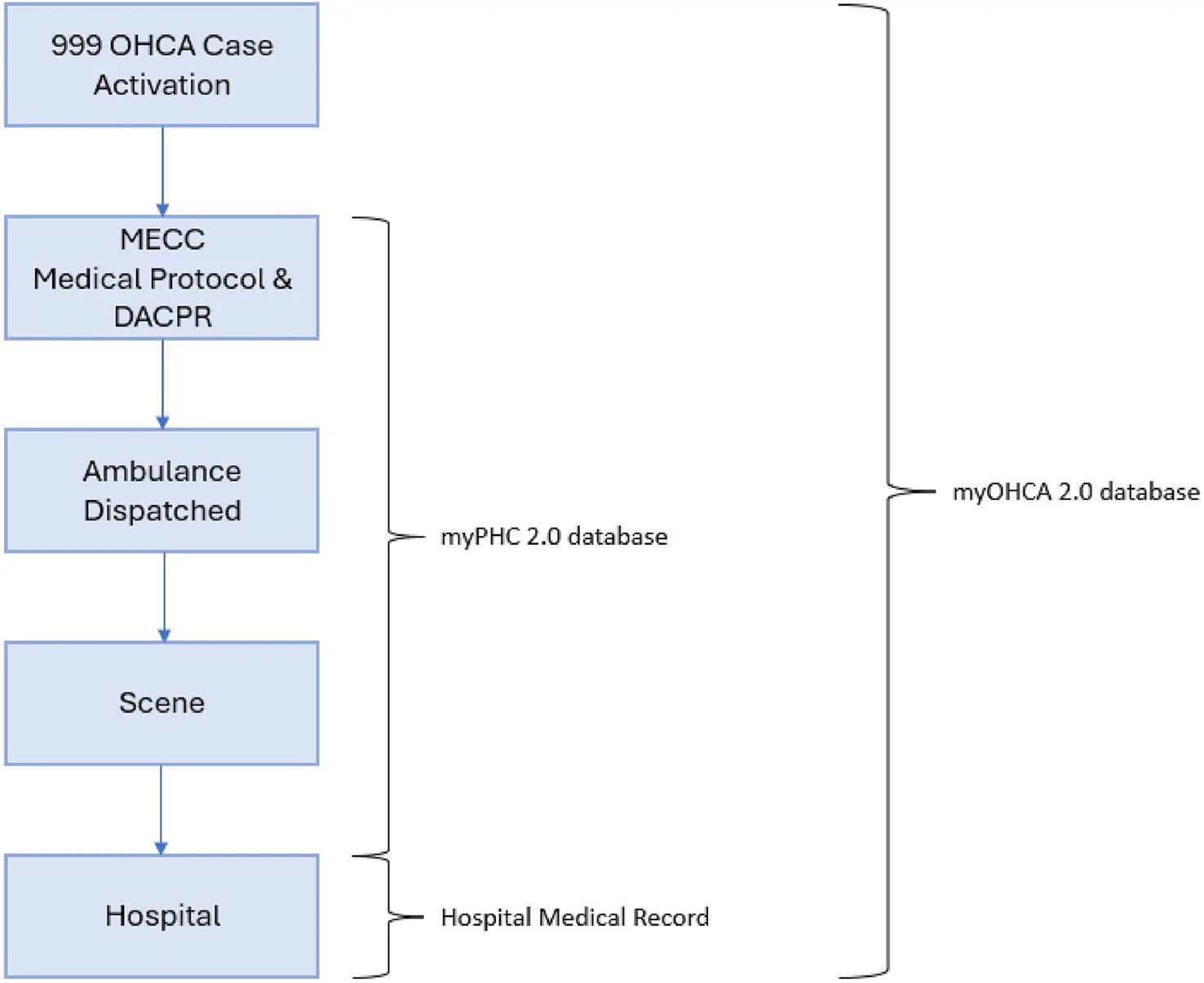
**EMS system coordination for OHCA response in Penang**. *Abbreviations:* out-of-hospital cardiac arrest (OHCA); Medical Emergency Coordination Centre (MECC); dispatcher-assisted cardiopulmonary resuscitation (DACPR).

**Fig. 2:**
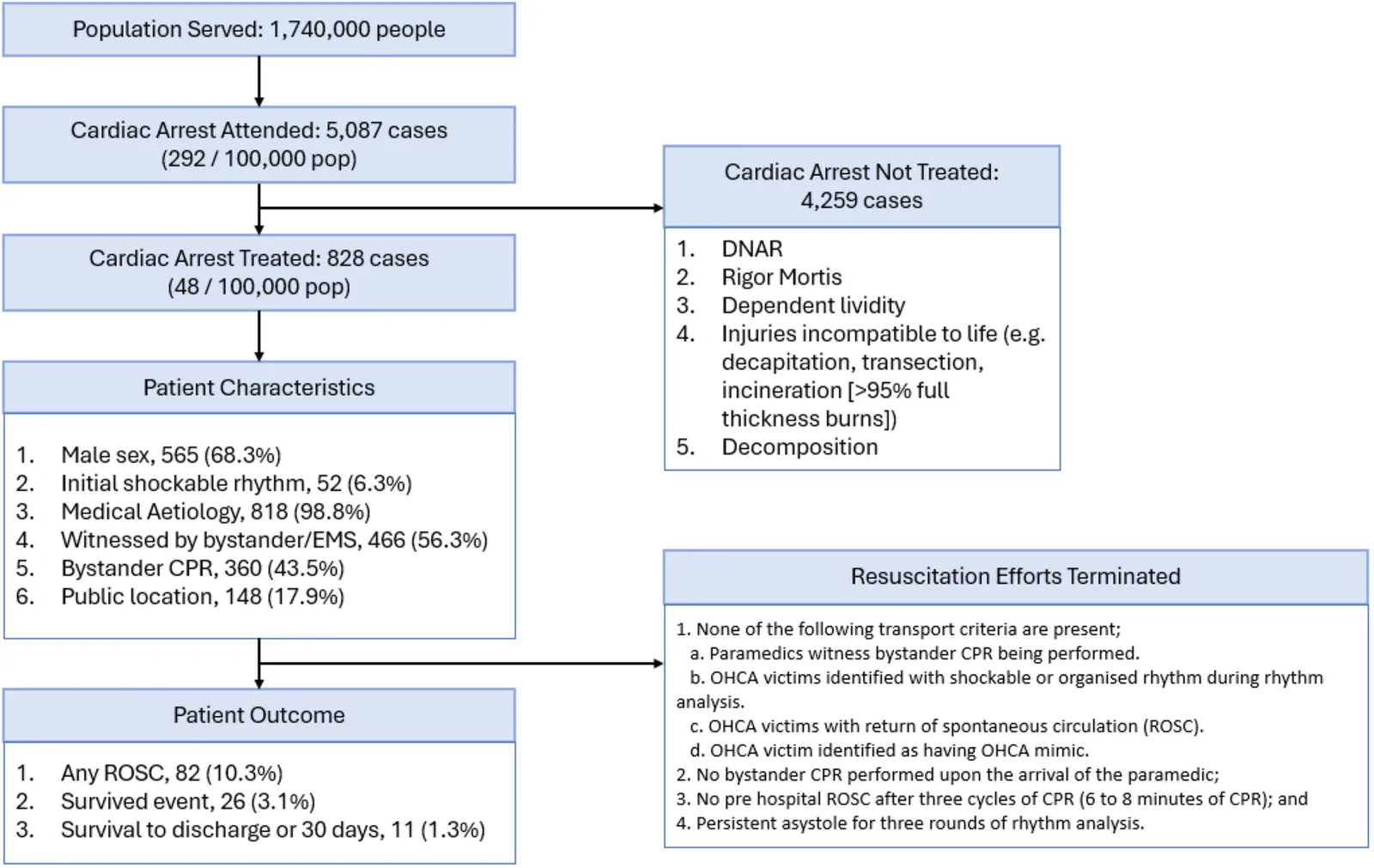
**Utstein-style patient flow diagram for OHCAs in Penang**. *Abbreviations:* do not attempt resuscitation (DNAR); cardiopulmonary resuscitation (CPR); return of spontaneous circulation (ROSC).

The state of Penang in Malaysia has a population of 1.74 million and a land area of 1039 km^2^. Penang EMS is a single-tier, hospital-based public service that dispatches ambulances from six government hospitals via the state Medical Emergency Coordination Centre (MECC), supplemented by non-government organisations and private hospitals. The service responds to all OHCAs occurring within the state using an electronic dispatch system (myPHC2.0) for real-time coordination between call dispatchers, paramedics (Assistant Medical Officers), and personnel from receiving hospitals (Fig. 1). Government paramedics are trained in advanced life support (ALS) and are permitted to provide manual defibrillation, administer adrenaline and amiodarone, and establish an advanced airway (via endotracheal tube or supraglottic airway). EMS personnel adhere to the Minimum Standards Required for the Management of Adult OHCA in Prehospital Care Services, published by the College of Emergency Physicians, Academy of Medicine Malaysia in 2016.^2^ Resuscitation is initiated for unconscious, apnoeic, or abnormal-breathing patients suspected of OHCA and continued if paramedics witnessed bystander CPR. Paramedics may terminate resuscitation under certain criteria (Fig. 2), then confirm absence of signs of life, notify family members, and inform authorities for death certification.

### Participants

The Penang OHCA registry includes all OHCA cases occurring in adult, paediatric, and neonatal patients who experienced cessation of cardiac function regardless of aetiology, and who are attended by Penang EMS at any stage during the event.

### Variables

Patient and event characteristics were recorded following the Utstein guidelines.^3^ Additional characteristics relevant to the Malaysian context (e.g., patient ethnicity) were also collected. The primary outcome was any EMS-documented return of spontaneous circulation (ROSC) before Emergency Department arrival. The secondary outcome was 30-day survival.

### Data collection

When OHCA is recognised by a dispatcher and confirmed by an on-scene paramedic, an incident record is created on myOHCA2.0. Data collected following the Utstein guidelines^3^ is populated from three sources: 9-9-9 call details auto-synced via myPHC2.0; paramedic-entered on-scene treatment or transport data; and in-hospital treatment and patient outcome entered by trained research assistants. The myOHCA2.0 platform utilises standardised data-entry fields and automated numeric validation to ensure accuracy and data completeness. Research assistants review each record before finalising it in the registry.

### Data analysis

Descriptive statistics were computed for patient, event, response, and outcome characteristics. OHCAs were stratified by whether or not EMS personnel attempted resuscitation, and groups were compared using Mann-Whitney U tests for continuous variables and chi-squared tests for categorical variables. All statistical tests were two-sided, with a *p*-value less than 0.05 deemed statistically significant. All missing data were acknowledged and excluded from analysis. Data analysis was conducted using SPSS Version 29 (IBM Inc., Armonk, NY, USA).

## Results

In total, 5087 OHCAs (292 per 100,000 person-years) were recorded by the Penang OHCA registry during the study period, of which EMS attempted resuscitation in 828 (16.3%) (Fig. 2). Table 1 displays descriptive characteristics of the included OHCAs stratified by whether or not EMS attempted resuscitation. The recorded OHCA cases were predominantly male (54.8%) with a median age of 73 (IQR 61–83) years. Compared to untreated patients, EMS-treated patients were more likely to be male and younger compared to untreated patients, and their arrests were more likely to have been witnessed by bystanders (47.2% vs 34.6%) or EMS personnel (9.1% vs 1.2%). The median EMS response time was 22 min [IQR 16–30]. The majority of patients were of either Chinese (52.2%) or Malay (33.0%) ethnicity and experienced their OHCA in homes (92.8%). EMS-treated OHCAs had bystander CPR performed in 43.5% of cases and an automated external defibrillator (AED) applied in 7.1% of cases. Over half of OHCAs with attempted resuscitation by EMS (60.1%) were transported to hospital, with return of spontaneous circulation and 30-day survival occurring in 10.3% and 1.3% of cases, respectively.

**Table 1:** Characteristics of included OHCA cases.

**Variable**	**All records** **(*N* = 5087)**	**OHCA with EMS resuscitation attempt** **(*n* = 828)**	**OHCA without EMS resuscitation attempt** **(*n* = 4259)**	***p* value**	**Missing, *n* (%)**
Age in years, median [IQR]	73 [61–83]	63 [49–75]	75 [64–84]	<0.001	29 (0.6)
Aged <18 years, *n* (%)	33 (0.7)	17 (2.1)	16 (0.4)	<0.001	
Sex, *n* (%)				<0.001	6 (0.1)
Male	2785 (54.8)	565 (68.3)	2220 (52.2)		
Female	2296 (45.2)	262 (31.7)	2034 (47.8)		
Ethnicity, *n* (%)				<0.001	36 (0.7)
Chinese	2639 (52.2)	351 (42.6)	2288 (54.1)		
Malay	1666 (33.0)	311 (37.7)	1355 (32.1)		
Indian	499 (9.9)	116 (14.1)	383 (9.1)		
Other	247 (4.9)	46 (5.6)	201 (4.8)		
Public location, *n* (%)	365 (7.2)	148 (17.9)	217 (5.1)	<0.001	1 (0.0)
Time of day, *n* (%)				0.789	4 (0.1)
Daytime (08:00–15:59)	2026 (39.9)	321 (38.8)	1705 (40.1)		
Evening (16:00–23:59)	1700 (33.4)	283 (34.2)	1417 (33.3)		
Overnight (00:00–07:59)	1357 (26.7)	223 (27.0)	1134 (26.6)		
Day of week, *n* (%)				0.345	0 (0.0)
Weekday (Monday-Friday)	3596 (70.7)	572 (69.3)	3022 (71.0)		
Weekend (Saturday-Sunday)	1491 (29.3)	254 (30.7)	1237 (29.0)		
Witnessed arrest, *n* (%)				<0.001	0 (0.0)
By bystander	1866 (36.7)	391 (47.2)	1475 (34.6)		
By EMS personnel	126 (2.5)	75 (9.1)	51 (1.2)		
Bystander CPR performed, *n* (%)	397 (7.8)	360 (43.5)	37 (0.9)	<0.001	0 (0.0)
Bystander AED Applied, *n* (%)	125 (2.5)	59 (7.1)	66 (1.5)	<0.001	0 (0.0)
EMS response time in minutes, median [IQR]	21 [16–29]	22 [16–30]	22 [16–29.5]	0.988	37 (0.7)
Initial shockable rhythm detected by EMS, *n* (%)	52 (1.0)	52 (6.3)	0 (0.0)	<0.001	0 (0.0)
Mechanical CPR applied, *n* (%)	95 (1.9)	95 (11.5)	0 (0.0)	<0.001	0 (0.0)
Transported to hospital, *n* (%)	497 (9.8)	497 (60.1)	0 (0.0)	<0.001	1 (0.0)
Return of spontaneous circulation, *n* (%)	84 (1.7)	82 (10.3)	0 (0.0)	<0.001	65 (1.3)
Survived event, *n* (%)	26 (3.1)	26 (3.1)	0 (0.0)	<0.001	23 (0.5)
Survival to 30 days, *n* (%)	11 (0.2)	11 (1.3)	0 (0.0)	<0.001	11 (0.2)

## Discussion

This study demonstrated a low rate of survival among OHCA patients in Penang, Malaysia. Importantly, only 16.3% of all OHCA cases received an EMS resuscitation attempt. This is largely attributable to the overlap between OHCA and “confirm death” calls, as 999 is activated for both types of calls, and paramedics do not initiate resuscitation if obvious signs of death are present upon arrival as per treatment standards.^2^ Among EMS-treated cases, poor outcomes were likely driven by prolonged EMS response times and low rates of bystander CPR and AED use, each of which is known to affect survival.^4^, ^5^ These findings align with previous Malaysian and other low- and middle- income country (LMIC) studies reporting delayed response, low bystander intervention, and correspondingly poor outcomes.^4^, ^6^ Notably, Penang’s crude OHCA incidence during the study period (292 per 100,000 person-years) was more than four times Singapore’s (67 per 100,000 person-years),^7^ with a lower rate of bystander CPR (43.5% vs 59.4%). Given that most arrests occur at home (92.8%), interventions like mandatory school-based BLS training may be warranted. Furthermore, the median EMS response time of 22 min is suboptimal and particularly concerning. A study in the neighbouring country of Thailand found that response times under 6 min were associated with three-fold higher odds of survival to hospital discharge.^4^ These metrics represent clear, modifiable targets for quality improvement, and the registry provides a robust foundation for monitoring and refining interventions to improve OHCA outcomes in Penang.

The launch of the myOHCA2.0 platform enabled sustainable, systematic OHCA data collection in Penang. Previously, OHCA research relied on manual abstraction of data from existing care records^1^ into a Google Form, which was a labour-intensive process prone to inconsistencies and incomplete entries. The myOHCA2.0 platform overcame this via Utstein-aligned fields,^3^ automated electronic synchronisation and built-in validation rules to enhance accuracy. High-quality data are essential for evaluating interventions like dispatcher-assisted CPR, community CPR training, and evidence-driven ambulance deployment. Such registry infrastructure has proven value globally: the Pan-Asian Resuscitation Outcomes Study (PAROS) showed measurable survival gains in resource-limited settings through policy reform.^8^ Similarly, high-income registries such as CARES in the United States^9^ have used benchmarking and system gap analysis to implement evidence-based changes that substantially improved OHCA outcomes over time.

Finally, it is important to note that the functions of myOHCA2.0 can be easily programmed via open-source coding platforms. The system also leverages the Ministry of Health, Malaysia MyGovUC 3.0 platform, ensuring secure database access restricted to authorised personnel. Thus, costs for proprietary software, server infrastructure, and ongoing maintenance are significantly reduced, making the establishment of a sustainable OHCA registry plausible in resource-limited regions without sacrificing data quality. Prospects for international adoption of this universally integrable registry technology strongly align with the World Health Organisation (WHO) goals for LMIC, where OHCA underreporting exceeds 70%.^5^ Technological innovations such as myOHCA2.0 could bridge the gap between emergency care efficiency in LMICs and that of higher-income countries by supporting robust data collection, multilingual interfaces and even integration with mobile first-responder reporting tools. With more regions establishing OHCA registries, this could scale OHCA epidemiological surveillance globally and further cultivate collaborative research networks, strengthening global resuscitation developments beyond the traditional focus of high-income countries. Future research would need to evaluate long-term implementation and effectiveness in other settings.

## Limitations

As this study is retrospective in nature, it cannot infer causality, and the analysis is subject to confounding by unmeasured variables, including comorbidities and the in-hospital care process. The study is also limited by missing data, potentially introducing selection bias. Furthermore, data quality is dependent on accurate EMS documentation under time-critical conditions, and bystander interventions may be subject to recall or reporting bias.

## Conclusion

Penang’s OHCA survival rate remains low despite bystander CPR being performed in nearly half of all EMS-treated cases. Prolonged EMS response times and low rates of AED deployment is likely to contribute to low survival and are targets for intervention. The myOHCA2.0 platform has provided a sustainable, standardised data infrastructure needed to drive OHCA quality improvement initiatives in Malaysia.

## Sources of funding

None.

## CRediT authorship contribution statement

**K.E. Emily Leong:** Writing – original draft, Visualization, Software, Methodology, Investigation, Formal analysis. **K.H. Benjamin Leung:** Writing – original draft, Supervision, Methodology, Investigation, Data curation. **Tomas Barry:** Writing – review & editing, Validation, Supervision, Conceptualization. **Yuen Chin Leong:** Writing – review & editing, Visualization, Supervision, Software, Resources, Methodology, Conceptualization.

## Declaration of competing interest

The authors declare that they have no known competing financial interests or personal relationships that could have appeared to influence the work reported in this paper.

## Appendix A Supplementary material

Supplementary Data 1 Cleaned 1-year myOHCA2.0 registry raw data.
